# Evolutionary rise of a synaptic mechanism for creating and diversifying key reinforcement signals

**DOI:** 10.64898/2026.07.31.742134

**Published:** 2026-08-28

**Authors:** Natalia Rodriguez-Sosa, Lupita Rios, You-Hsin Lin, Volodymyr Rybalchenko, Yash Sharma, Alaa Hajeissa, Ian Chambers, Nien-Shao Wang, Raghav Rajesh, Vishal Narla, Steven Shabel

## Abstract

Most neurons release either excitatory or inhibitory neurotransmitters. However, multiple inputs to the lateral habenula (LHb) co-transmit glutamate and GABA, transmitters with opposing effects on LHb output. Although the LHb has an established role in reinforcement learning, the adaptive significance of glutamate/GABA co-release remains unclear. Using experimentally informed simulations, we show that GABA co-release is sufficient to produce temporal difference (TD)-like transformations of input activity, computations commonly used for reinforcement learning and behavioral optimization. Heterogeneous GABA-to-glutamate ratios, like those found among LHb neurons ex vivo, produce diverse TD-like computations linked to higher-order decision-making. Single-cell RNA-sequencing analysis and machine-learning image analysis further indicate that glutamate/GABA co-release expanded across vertebrate evolution, from fish to mice, rats, and monkeys. Evolutionary expansion of glutamate/GABA co-release may have supported increasingly sophisticated learning and decision-making that contribute to intelligent behavior.

## Full Text Availability

The license terms selected by the author(s) for this preprint version do not permit archiving in PMC. The full text is available from the preprint server.

